# Imaging of ankylosing spondylitis

**DOI:** 10.1186/ar3725

**Published:** 2012-03-08

**Authors:** Mikkel Østergaard

**Affiliations:** 1 Department of Rheumatology, Copenhagen University Hospital at Glostrup, Copenhagen, Denmark

## 

Conventional radiography can visualize bone erosion, sclerosis, joint space narrowing and new bone formation in sacroiliac joints and the spine, but is unfortunately not very sensitive in early disease. Diagnosis of ankylosing spondylitis (AS) is dependent on presence of bilateral moderate or unilateral severe radiographic sacroiliitis, as part of the modified New York criteria for AS. This has, until recently (see MRI below), delayed the diagnosis by 7-10 years. Furthermore, the modified Stokes ankylosing spondylitis spine score (mSASSS), which is the most sensitive radiographic method for monitoring structural damage in AS, is not very reproducible or sensitive to change, so improved methods for structural damage assessment are highly needed.

MRI has resulted in a major improvement in the evaluation and management of patients with SpA. Firstly, it permits earlier diagnosis, as MRI findings of active sacroiliitis form part of the recent ASAS criteria for axial spondyloarthritis. Secondly, MRI can provide objective evidence of currently active inflammation in patients with SpA. MRI is by far the best available method for detecting and monitoring inflammation in the spine and sacroiliac joints, and several validated assessment systems exist. Until the introduction of MRI, disease activity assessment was restricted to patient-reported outcomes, such as the Bath ankylosing spondylitis disease activity index (BASDAI) and functional index (BASFI), because disease activity could not be assessed in a sensitive manner by biochemical (mainly C- reactive protein (CRP)) or physical evaluation. Furthermore, MRI can visualize structural damage (erosion, fat infiltration, syndesmophytes and ankylosis) in the sacroiliac joint and spine, but the clinical role of MRI for monitoring structural damage remains to be established.

Finally, certain MRI findings (inflammation at the vertebral corners) have predictive value with respect to subsequent development of radiographic syndesmophytes. However, clarification of the prognostic value of MRI in clinical practice requires further research.

Despite contrast-enhanced Doppler US has been reported to have a high negative predictive value for the detection of sacroiliitis, the role of US in assessment of sacroiliac and spine involvement in AS and other types of axial SpA is minimal.

